# Characterization of two types of intranuclear hepatocellular inclusions in NAFLD

**DOI:** 10.1038/s41598-020-71646-y

**Published:** 2020-10-06

**Authors:** Suzan Schwertheim, Julia Kälsch, Holger Jastrow, Christoph Matthias Schaefer, Sarah Theurer, Saskia Ting, Ali Canbay, Heiner Wedemeyer, Kurt Werner Schmid, Hideo Andreas Baba

**Affiliations:** 1 Institute of Pathology, University Hospital of Essen, University of Duisburg-Essen, Hufelandstr. 55, 45147 Essen, Germany; 2 Department of Gastroenterology and Hepatology, University Hospital of Essen, University of Duisburg-Essen, Essen, Germany; 3 Institute of Anatomy and Electron Microscopy Unit of Imaging Center Essen, University Hospital of Essen, University of Duisburg-Essen, Essen, Germany; 4 Department of Medicine, Ruhr University Bochum, University Hospital Knappschaftskrankenhaus Bochum, 44892 Bochum, Germany; 5 West German Cancer Centre Essen (WTZE), Essen, Germany

**Keywords:** Liver diseases, Hepatology, Cancer, Cell biology, Gastroenterology

## Abstract

Nuclear inclusions (NI) are a common finding in hepatocytes from patients with liver disease especially in diabetes mellitus and non-alcoholic fatty liver disease (NAFLD) but studies examining the shape and content of these inclusions in detail are lacking. In this study we define two distinct types of NI in NAFLD: inclusions bounded by the nuclear membrane, containing degenerative cell organelles and heterolysosomes (type1) and inclusions with deposits of glycogen but without any kind of organelles and delimiting membrane (type2). NI in 77 paraffin-embedded patients of NAFLD including NAFL and non-alcoholic steatohepatitis (NASH) were analyzed. In 4–12% of type1 NI immunopositivity for the autophagy-associated proteins LC3B, ubiquitin, p62/sequestosome1, cathepsin D and cathepsin B were detected with co-localizations of ubiquitin and p62; type2 NI showed no immunoreactivity. Three-dimensional reconstructions of isolated nuclei revealed that NI type1 are completely enclosed within the nucleus, suggesting that NI, although probably derived from cytoplasmic invaginations, are not just simple invaginations. Our study demonstrates two morphologically different types of inclusions in NAFLD, whereby both gained significantly in number in advanced stages. We suggest that the presence of autophagy-associated proteins and degenerated organelles within type1 NI plays a role in disease progression.

## Introduction

The most common chronic liver disease in Western countries is non-alcoholic fatty liver disease (NAFLD), affecting up to 30% of the adult population^1^. NAFLD is already considered as the hepatic manifestation of the metabolic syndrome^2^. Both clinically and histologically, NAFLD shows a broad spectrum of disease variety. Its spectrum embraces simple steatosis in non-alcoholic fatty liver (NAFL) to advanced non-alcoholic steatohepatitis (NASH), which may be associated with fibrosis and progression to cirrhosis or hepatocellular carcinoma (HCC)^3,4^. Morphologic alterations in NAFLD might not only alter the cytoplasm but also the nucleus. Nuclear vacuolation in hepatocytes was first reported over a century ago by Paul Ehrlich^5^. He showed glycogen filled vacuoles in nuclei with eccentrically displaced chromatin in liver tissue obtained from autopsies of diabetic patients. When stained with haematoxylin and eosin (HE) glycogen was leached out and these formations appeared as an empty nucleus^5^. These so called glycogenated nuclei are found in up to 75% in non-alcoholic steatohepatitis (NASH)^6^. On the ultrastructural level these “vacuoles” are filled with typical glycogen granules with diameters of 3–7 nm and are not delimited by a membrane^7^. In subsequent studies, nuclear vacuolation was described in different liver diseases, such as non-alcoholic fatty liver disease (NAFLD), alcoholic steatohepatitis or Wilson’s disease^8–12^.

Intranuclear inclusions (NI) have been studied for over 100 years^13^. They have been considered to have a homogenous morphology until Cowdry et al. were the first to divide these NI in patients with viral infections into two groups depending on alterations of cell morphology as a reaction to a virus, the cowdry bodies^14,15^. These early studies were based on mere description of morphological aspects of inclusions in light microscopy. Later, in vivo studies demonstrated the induction of NI by injections of colchicine or thioacetamide in mouse hepatocytes^16,17^. Ultrastructural characterization of NI in mouse liver and HCC revealed inclusions bounded by a double membrane originating from the nuclear envelope^18^.

In NAFLD, nuclear vacuolation is linked to aging process or senescence but no differentiation is made between NI with or without a limiting membrane^19^. Our recent study of membrane-bounded NI in HCC^20^ and in thyroid carcinomas^21^ has shown the presence of autophagy-associated proteins and proteases within the inclusions; in HCC the occurrence of inclusions was associated with patients’ survival benefit^20^. Jaskolski et al. described that autophagy is involved in the pathogenesis of intranuclear vacuolation in meningeomas^22^; their electron microscopic studies revealed lysosomal bodies and autophagic vacuoles within these NI, suggesting an active macroautophagy process^22^.

Thus, the aim of our current study was to characterize NI in NAFLD morphologically in detail and to clarify a possible association with autophagy and disease progression. Therefore, we analyzed the NI found in our NAFLD cohort first on the presence of a limiting membrane and secondly on storage of glycogen. Staining with periodic acid-Schiff (PAS) gave proof of glycogen infiltration. We performed three-dimensional (3D) reconstructions of isolated hepatocyte nuclei to clarify the shape of the NI; transmission electron microscopy (TEM) studies were used to analyze the NI regarding limiting membrane and content in more detail. To investigate if these inclusions might have a biological function we further examined them with immunohistochemistry (IHC) for the presence of the autophagy-associated proteins p62/sequestosome1, ubiquitin, LC3B, cathepsin B and cathepsin D^23^ whereby p62 is involved, among others, in NFκB^24,25^ and Keap1-Nrf2^26^ signaling pathways. Three-dimensional reconstruction of double immunofluorescence (IF) -labelled tissue sections was performed to elucidate the exact spatial location of the inclusions and accumulated proteins.

## Results

### Occurrence of two different types of NI in NAFLD

**Figure 1:**
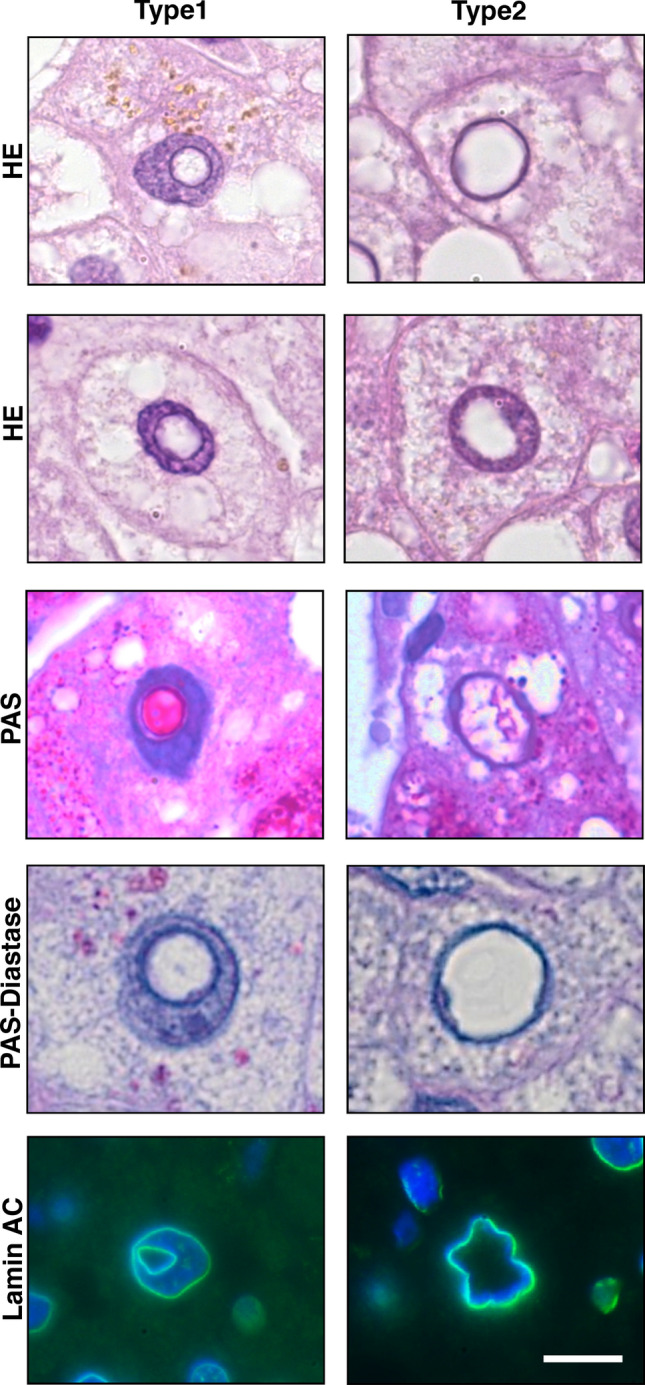
Characterization of type1 and type2 NI in NAFLD. The images show two representative HE-stainings of tissue sections from type1 and type2 NI demonstrating that type1 NI are surrounded by a basophilic membrane, which is lacking in type2 NI. Positive PAS staining is detected both for type1 and type2 NI and the negative staining for PAS diastase depicts glycogen accumulation as the origin for this positivity. Immunofluorescence analysis on lamin AC expression demonstrates that lamin AC is at the membrane bordering type1 NI. The white bar at the bottom right, representative for all images indicates 10 µm. Original magnifications: 1,000 X.

Analysis of HE sections in the 58 morbidly obese patients and 19 organ donor patients (control group) revealed NI in 76 out of the 77 cases. The one case in which we did not detect any NI at all was in the control group. These NI were different in shape and size (Fig. 1). The most obvious difference was the presence of a membrane around some NI. We designated these NI as “type1” (left column of Fig. 1). Whereas all the inclusions’ internal area appeared empty or heterogeneous in HE staining, type1 NI were limited by a basophilic membrane. However, the inclusions we called as “type2” NI were not surrounded by a basophilic membrane; these inclusions are also known as glycogenated nuclei^5,6^ (right column of Fig. 1). For both types the staining reaction of the inclusions’ internal area was often similar to that of the cytoplasm but differed from that of the karyoplasm. We detected in both types of NI accumulation of glycogen. Lamin AC immunofluorescence analysis demonstrated clearly the difference between both types of inclusions: type1 inclusion were bounded by a membrane with lamin AC immunopositivity, whereby type2 inclusions appeared to be either surrounded by no membrane at all or at least by no membrane that was positive for lamin AC (Fig. 1). Since lamin AC is a nuclear membrane marker, our discovery of lamin AC at the membrane bordering type1 inclusions, suggests its nuclear membrane origin.

### Correlation of the presence of type1/type2 NI and NAFLD disease severity

**Figure 2:**
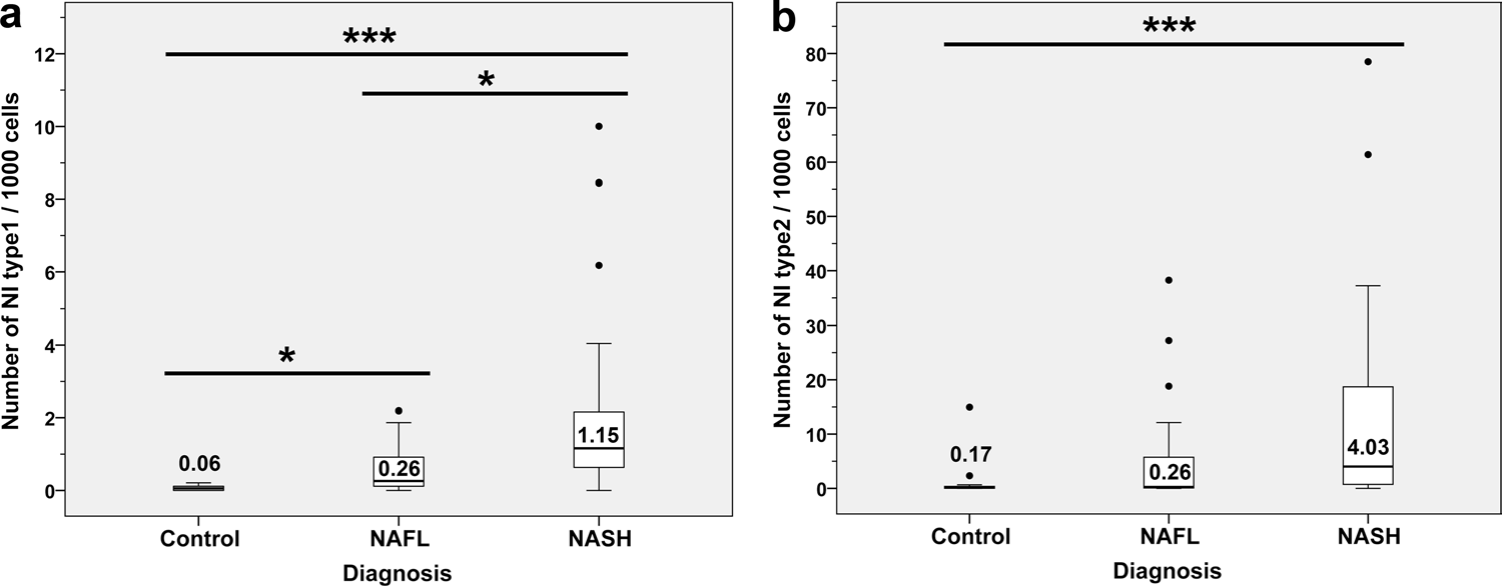
Associations between the number of NI and disease progression. (**a**) Number of type1 NI/1,000 cells increases with progression of NAFLD; NASH vs. controls: ***P < 0.001; NASH vs. NAFL: *P = 0.012; NAFL vs. controls: *P = 0.016. Number of type1 NI/1,000 cells: Control 0.06 (0–0.21), NAFL 0.26 (0–2.19), NASH 1.15 (0–10). (**b**) Type2 NI/1,000 cells are more in NASH than in controls: ***P = 0.001. Number of type2 NI/1,000 cells: Control 0.17 (0–14.96), NAFL 0.26 (0–38.25), NASH 4.03 (0–78.49); values are medians with ranges presented in parentheses. (All Kruskal–Wallis test; bold lines inside the box plot represent median levels; the values are shown). Results are significant at *P ≤ 0.05 and ***P ≤ 0.001.

Additionally, NI were studied quantitatively. The total cell count within the whole tissue section for each case was performed by automatic counting identifying nuclei by positive DAPI staining. Then from the same paraffin block a HE stained tissue section was analyzed on the number of type1 and type2 NI. We counted the number of NI with (type1) or without (type2) delimiting basophilic membrane within the whole section. If a nucleus contained more than one intranuclear inclusion, we counted this as one intranuclear inclusion. To eliminate the bias effect of different tissue size, we divided the number of NI by the total cell count and multiplied it with factor 1,000 for more convenience. Details are depicted in Fig. 2 showing the median values for the number of NI type1 (Fig. 2A) and type2 (Fig. 2B) per 1,000 cells. The median number of type2 NI/1,000 cells in NASH was with 4.03 (range: 0–78.5) 3.5 times higher than that of type1 NI/1,000 cells with 1.15 (range: 0–10). Number of valid cases was 19 Control, 23 NAFL and 35 NASH cases. To investigate whether there was a correlation between the occurrence of NI and NAFLD, we performed the Kruskal–Wallis test. Interestingly, both membrane-bounded NI (type1) and vacuolated nuclei i.e. non-membrane bounded NI (type2) were significantly more in NASH compared to controls (*P* ≤ 0.001; Fig. 2A,B). Additionally, the number of type1 NI was significantly higher in NASH than in NAFL (*P* = 0.012; Fig. 2A) and type1 NI were significantly more in NAFL than in controls (*P* = 0.016; Fig. 2A). Study of Spearman's rho correlations showed significant correlations between type1/type2 NI and disease stages. We detected for both type1 NI (*P* < 001; rho = 0.659) and type2 NI (*P* < 001; rho = 0.428) strong associations with NAFLD progression; this correlation was slightly stronger for type1 NI than for type2 NI.

### Studies regarding correlations between clinical/laboratory parameters and the number of type1/type2 NI

We have classified fasting blood glucose into three levels: normal range: 70–99 mg/dl, pre-diabetes: 100–125 mg/dl; and diabetes: ≥ 126 mg/dl according to the criteria of the American Diabetes Association (ADA)^27^. Then we split the patients into two groups: we combined the patients with pre-diabetes and diabetes into one group; in the second group were all patients with normal glucose levels. We found a significantly higher number of NI type1 in the pre-diabetes and diabetes group than in the normal group (*P* = 0.032); details are shown in Table 1. Study of Spearman's rho correlations showed significant correlation between the number of NI type1 and fasting glucose levels (*P* = 0.043; rho = 0.276). Additionally, we analyzed the data on an association between elevated cholesterol, triglyceride, AST and GGT levels and the number of NI type1 and 2. We detected a significant association between elevated triglyceride levels and the number of NI type1 (*P* = 0.006) and NI type2 (*P* = 0.001). Further, patients with elevated GGT levels had significant more NI type2 (*P* = 0.050). Also the association between the number of NI type1/2 and the steatosis, ballooning and lobular inflammation grades was significant (*P* < 0.001, Table 1).

**Table 1:** Correlation of clinical/laboratory parameters with the number of NI type1/type2.

Parameters	n valid cases	Number of NI type1		Number of NI type2	
		Median value^b^	*P* value*	Median value^b^	*P* value*
**Fasting glucose (mg/dl)**					
Normal (70–99)	19	0.42 (0–1.98)	0.032	0.35 (0–27.23)	0.068
Pre-diabetes and diabetes^a^	35	1.12 (0–8.46)		4.03 (0–78.49)	
**Total cholesterol (mg/dl)**					
Normal (< 200)	20	0.87 (0–8.46)	0.598	4.1 (0–61.39)	0.730
Elevated (> 200)	13	1.12 (0.26–3.79)		5.9 (0–78.49)	
**Triglyceride (mg/dl)**					
Normal (< 200)	18	0.66 (0–2.32)	0.006	0.8 (0–31.04)	0.001
Elevated (> 200)	7	2.33 (0.7–8.46)		27.2 (10.4–78)	
**ALT (U/L)**					
Normal (< 35/ < 50)	40	0.59 (0–8.46)	0.078	0.73 (0–61.4)	0.444
Elevated (> 35/ > 50)	24	1.05 (0–6.18)		2.73 (0–78.5)	
**AST (U/L)**					
Normal (< 35/ < 50)	28	0.59 (0–8.46)	0.617	0.33 (0–61.4)	0.204
Elevated (> 35/ > 50)	16	1.05 (0–6.18)		6.09 (0–78.5)	
**GGT (U/L)**					
Normal (< 35/ < 55)	42	0.61 (0–6.18)	0.134	0.37 (0–61.4)	0.050
Elevated (> 35/ > 55)	22	0.92 (0–8.46)		4.2 (0–78.5)	
**Fibrosis grade**					
0	10	1.15 (0–2.19)		0.55 (0–38.2)	
1	16	0.61 (0–2.32)		0.95 (0–31)	
2	28	0.96 (0–10)	0.179	4.8 (0–78.5)	0.159
3	4	0.48 (0–1.3)		2.04 (0–8.67)	
**Steatosis grade**					
0	19	0.06 (0–0.21)		0.17 (0–14.96)	
1	28	0.6 (0–2.19)		0.4 (0–38.25)	
2	18	1.17 (0–8.43)	< 0.001	2.25 (0–61.39)	< 0.001
3	12	1.99 (0.61–10)		12.8 (0.1–78.5)	
**Ballooning grade**					
0	37	0.11 (0–1.86)		0.17 (0–27.23)	
1	21	1.15 (0–3.53)	< 0.001	1.88 (0–61.39)	< 0.001
2	19	1.67 (0.21–10)		8.67 (0.1–78.5)	
**Lobular inflammation grade**					
0	35	0.11 (0–2.19)		0.21 (0–38.25)	
1	16	1.05 (0–8.46)		1.51 (0–37.24)	
2	24	1.47 (0–10)	< 0.001	4.06 (0–78.49)	0.004
3	2	0.44 (0.28–0.6)		1.8 (0.76–2.84)	

^a^We defined fasting blood glucose level of 100–125 mg/dl as pre-diabetes and glucose levels of ≥ 126 mg/dl as diabetes.

^b^Values are median number of type1/2 NI per 1,000 cells with ranges presented in parentheses. ALT and AST threshold for normal values were < 35 U/l for females and < 50 U/l for males; GGT threshold for normal values were < 35 U/l for females and < 55 U/l for males.

*P values correspond to the analysis of correlation between clinical/laboratory parameters with the number of type1/type2 NI in the HCC cohort. Mann–Whitney U-test and Kruskal–Wallis-tests were used for statistical analysis of the difference between two or more groups. Results are significant at *P* ≤ 0.05.

### Ultrastructural depiction of NI

**Figure 3:**
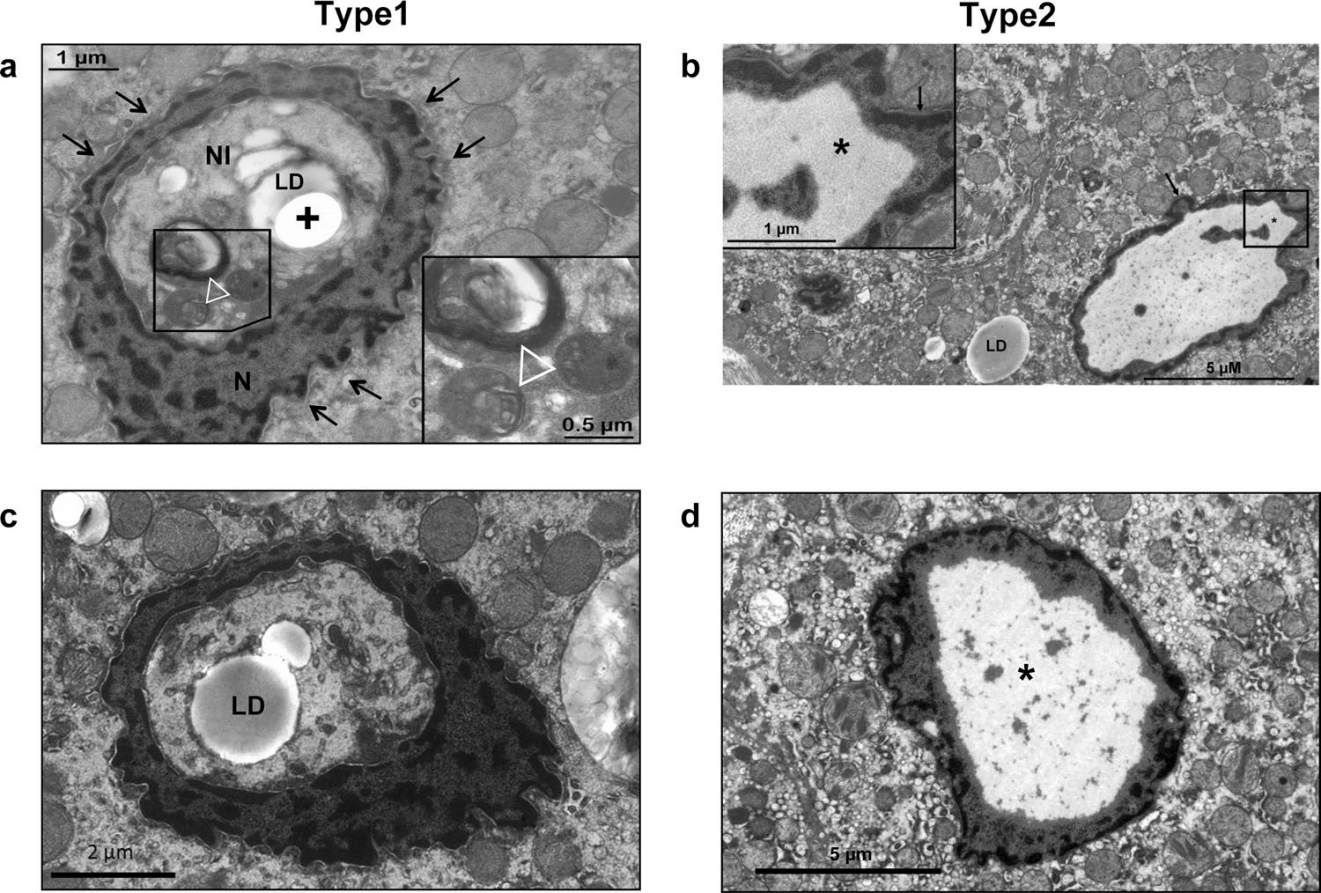
Ultrastructural analysis of type1 and type2 NI. (**a**) TEM image of a type1 intranuclear inclusion (NI) in NAFLD shows a hepatocyte nucleus (N) with NI coated by a membrane resembling the double-layer nuclear membrane (arrows). The NI contains three (hetero-) lysosomes (triangle), a lipid droplet (LD) and an artificial hole (+) in the sample. (**b**) The image reveals a type2 NI with deposit of glycogen (*). The nuclear membrane (arrow) delimiting the nucleus is shown and a lipid droplet (LD) within the cytoplasm. (**c**,**d**) The images depict additional examples of a type1 NI (**c**) and a type2 NI (**d**). Ultrathin sections were mounted on copper grids, double-stained with uranyl acetate (1%) and lead citrate (0.4%). Electron microscopy scale bars: Image A: 1 µm and 0.5 µm (in increased magnification); Image B: 5 µm and 1 µm (in increased magnification); Image C: 2 µm; Image D: 5 µm.

To investigate NI in NAFLD in more detail, we performed transmission electron microscopy (TEM) and observed that these NI were filled differently (Fig. 3). One type of intranuclear inclusion was coated by a membrane resembling the double-layer nuclear membrane (Fig. 3A,C). Our type1 membrane-bordered NI were filled with degenerated cell organelles and heterolysosomes. The content of the NI appeared to be more compressed compared to the cytoplasm (Fig. 3A,C). We detected membrane-limited NI both with invaginations of surrounding cytoplasm (data not shown) and totally lacking contact to the cytoplasm. In contrast to this our type2 NI had no additional membrane and appeared to be an “empty” nucleus with deposits of glycogen and did not contain any kind of cell organelles (Fig. 3B,D).

### 3D nuclear imaging: membrane-bounded NI are completely separated from the cytoplasm and closed

**Figure 4:**
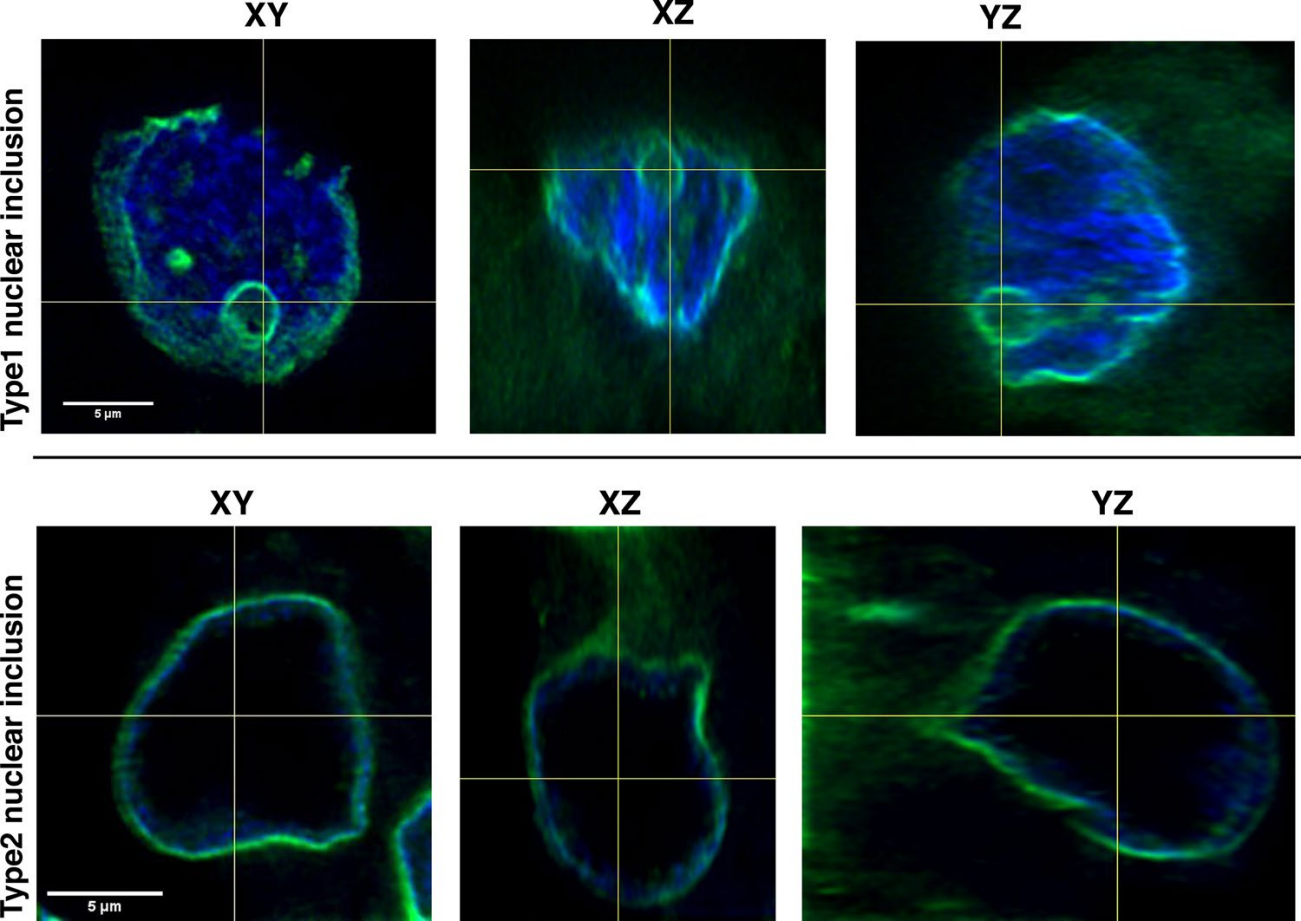
Three-dimensional (3D) reconstruction of immunofluorescence-labelled (lamin AC) isolated cell nuclei in X-, Y- and Z-axes. The images reveal representative cases with type1 and type2 NI. For the type1 intranuclear inclusion 161 optical sections of 0.1 µm were imaged and 113 of them were used for 3D reconstruction. The type2 intranuclear inclusion was reconstructed from 173 of 213 optical Sects. (0.1 µm). The type1 intranuclear inclusion is limited by a lamin AC (nuclear membrane marker) immunopositive membrane. The inclusion is completely closed in all planes and there is no DAPI (blue) staining within it. Lamin AC-positive immunostaining (light green) is also present in the nuclear membrane. The images of the type2 intranuclear inclusion in the bottom row show only immunopositivity for lamin AC in the nuclear membrane while the nucleoplasm appears “empty” in all planes.

We performed 3D reconstruction of isolated hepatocyte nuclei and stained them with immunofluorescence labelled lamin AC to examine the shape and the origin of the NI. We detected that type1 NI were delimited by a lamin AC stained intact membrane and were completely closed in all studied section planes (Fig. 4). There was no connection between the intranuclear inclusion and the cytoplasm. As the membrane bounding the inclusion was lamin AC positive, this indicates an origin from the nuclear membrane. Further, we demonstrate that the type1 intranuclear inclusion was completely enclosed within the nucleus (Fig. 4, Supplementary Movie 1, Supplementary Digital Content 1). In contrast, analysis of the type2 intranuclear inclusion showed no lamin AC stained membrane limitation (Fig. 4, Supplementary Movie 2, Supplementary Digital Content 2).

### Type1 NI contain autophagy-associated proteins

**Figure 5:**
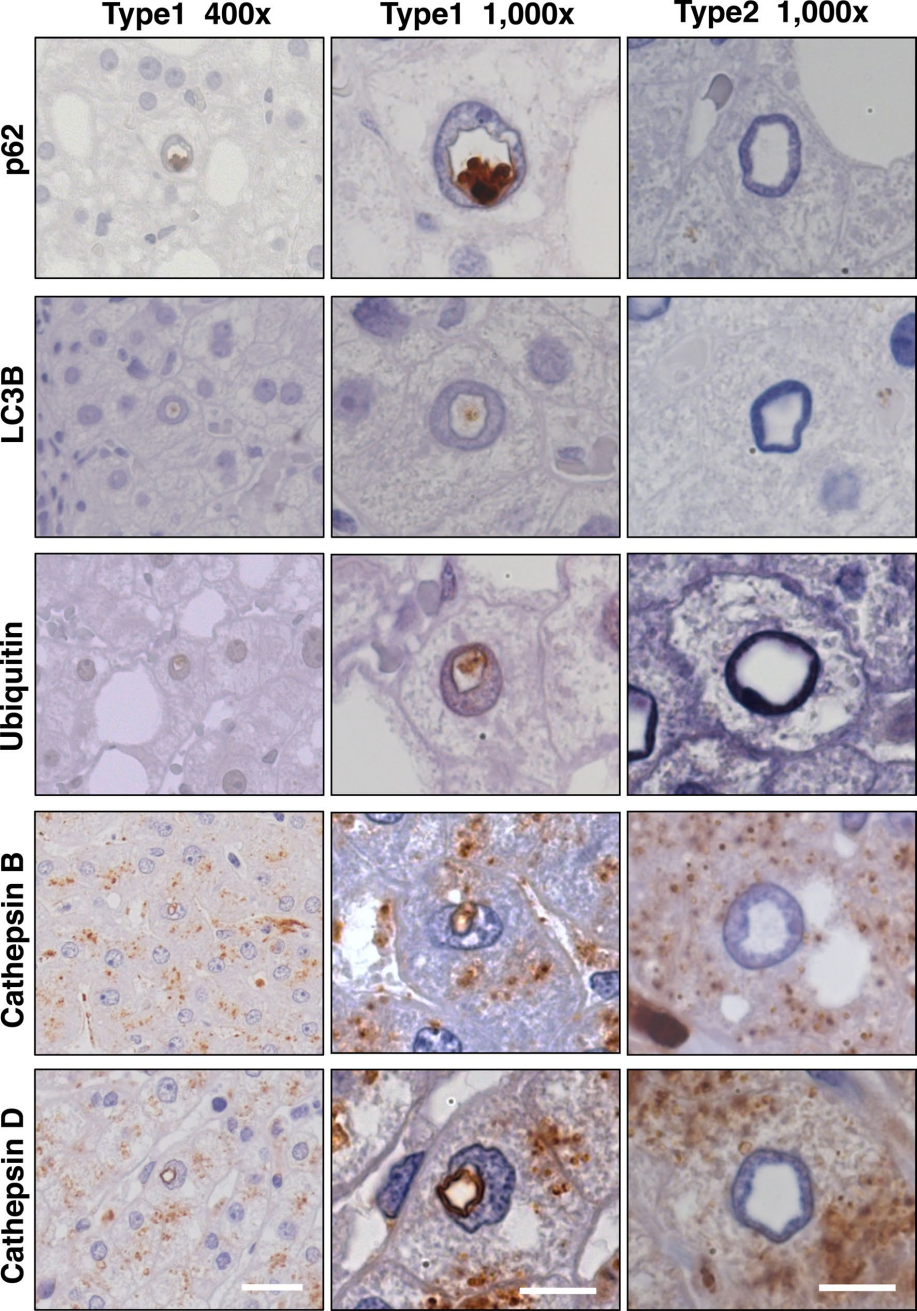
Immunohistochemical analysis for autophagy-associated proteins in type1 and type2 NI. Images depict positive immunoreactivity (brown) for autophagy-associated proteins within type1 NI, whereby type2 NI are all immunonegative. Analysis of type1 NI demonstrates strong accumulation of p62, LC3B, ubiquitin, cathepsin B and cathepsin D within the inclusions. Regarding p62, LC3B and ubiquitin this immunopositivity is almost exclusively within the inclusions and nearly lacking in the cytoplasm. Examination of type2 NI shows that these inclusions do not contain any autophagy-associated proteins. The white bar at the bottom left indicates 20 µm and the white bars at the bottom middle and right equals 10 µm. Original magnifications: 400 X (left) and 1,000 X (right).

Using immunohistochemistry, we examined whether there is a relationship between the manifestation of NI and autophagy. p62, ubiquitin, LC3B, cathepsin B and cathepsin D immunohistochemistry was performed in all 77 cases (Fig. 5). One case did not have any NI, therefore the total number of cases was 76. NI, which showed positive immunostaining for these autophagy-associated proteins, were counted individually. Most remarkably, none of the NI without a limiting membrane (type2) showed any positive immunostaining within the inclusions; instead these inclusions remained “empty” (right column of Fig. 5). We detected immunoreactivity for the autophagy-associated proteins exclusively in the membrane-bounded NI (type1). For quantitative evaluation of the results, first we focused on the HE stainings of the cases and counted the number of type1 and type2 NI. Thus, for all sections, a total sum of 6,618 NI without a limiting membrane (type2) and 959 membrane-bounded NI (type1) were counted. The total sum of immunopositive NI was for p62: 72 NI (72/959; 7.5%), for LC3B: 35 NI (35/959; 3.6%), for ubiquitin: 52 NI (52/959; 5.4%), for cathepsin B: 120 NI (120/959; 12.5%) and for cathepsin D: 96 NI (96/959; 10%), shown also in the Supplementary Table 1 (Supplementary Digital Content 3). We detected an accumulation of these proteins within NI; regarding p62, LC3B and ubiquitin, the immunostaining intensity was stronger in the NI compared to the surrounding cytoplasm.

**Table 2:** Clinical and laboratory data of the study groups.

Characteristics	Control (n = 19)	NAFL (n = 23)	NASH (n = 35)	n valid cases	*P* value*
Age (years)	45 (1–80)	38 (24–67)	45 (20–67)	23/35	0.242
Gender (male/female)	10/9	4/19	14/21	23/35	0.087
BMI (kg/m^2^)	23 (13–44)	49.9 (29.4–66.9)	52.9 (27.4–78.2)	21/33	0.231
Adiponectin (µg/mL)	–	3.1 (1.3–8.3)	2.95 (0.83–11.9)	21/25	0.991
CK18 M30 (IU/L)	–	184.9 (61.8–807.7)	367.6 (80.1–1,573.9)	21/25	0.001
CK18 M65 (IU/L)	–	333.4 (87.9–960.1)	636.7 (255.8–5,273.1)	20/25	< 0.001
Fasting glucose (mg/dl)	–	96.00 (73–150)	126 (72–385)	23/31	0.001
Total cholesterol (mg/dl)	–	198 (120–261)	177.5 (116–247)	15/18	0.320
Triglyceride (mg/dl)	–	149 (34–218)	207 (55–421)	13/12	0.041
ALT (U/L)	52 (9–3,272)	21 (13–65)	39 (14–120)	23/33	< 0.001
AST (U/L)	86 (20–3,078)	23 (16–49)	34 (23–90)	15/21	< 0.001
GGT (U/L)	135 (0–743)	20 (1.5–93)	41 (14–1,213)	23/33	< 0.001
**Fibrosis grade**					
0/1	–	4/7	6/9		
2/3	–	10/2	18/2	23/35	0.79
**Steatosis grade**					
0/1	19/0	0/20	0/8		
2/3	0/0	3/0	15/12	23/35	< 0.001
**Ballooning grade**					
0/1	19/0	18/3	0/18		
2	0	2	17	23/35	0.002
**Lob. Inflam. grade**					
0/1	19/0	16/4	0/12		
2/3	0/0	3/0	21/2	23/35	< 0.001

Values are medians with ranges presented in parentheses. *P values correspond to the comparison of NAFL / NASH and n valid cases reports the number of valid NAFL/NASH cases used for the statistical analysis. Statistic tests used were Mann–Whitney-U-tests for continuous factors and two-sided Fisher’s exact test for categorical parameters. *P* ≤ 0.05 was defined as statistically significant.

n, number; NAFL, non-alcoholic fatty liver; NASH, non-alcoholic steatohepatitis; ALT, alanine aminotransferase; BMI, Body Mass Index; AST, aspartate aminotransferase; GGT, gamma-glutamyl transferase; CK18, Cytokeratin18; Lob. Inflam. Grade, lobular inflammation grade.

Further, we examined correlations between disease progression and the number of type1 NI with positive immunoreactivity for the autophagy-associated proteins. Since we had shown in Fig. 2 that the number of type1 NI was significantly higher in NASH than in the controls, we related the respective total sum of immunopositive type1 NI in the individual disease groups to the total sum of type1 NI in the individual disease groups. We observed in 2.8–23.8% of the type1 NI immunopositivity for autophagy-associated proteins. However, since the number of type1 NI in the individual groups was different, a comparison of these groups regarding what percentage of these NI are immunopositive remains problematic. Therefore, only a descriptive study was possible. Details are listed in the Supplementary Table 1 (Supplementary Digital Content 3). Interestingly, there was an accumulation of autophagy-associated proteins in NI and these accumulations were only found in type1 NI.

### Presence of co-localized autophagy-associated proteins within type1 NI

**Figure 6:**
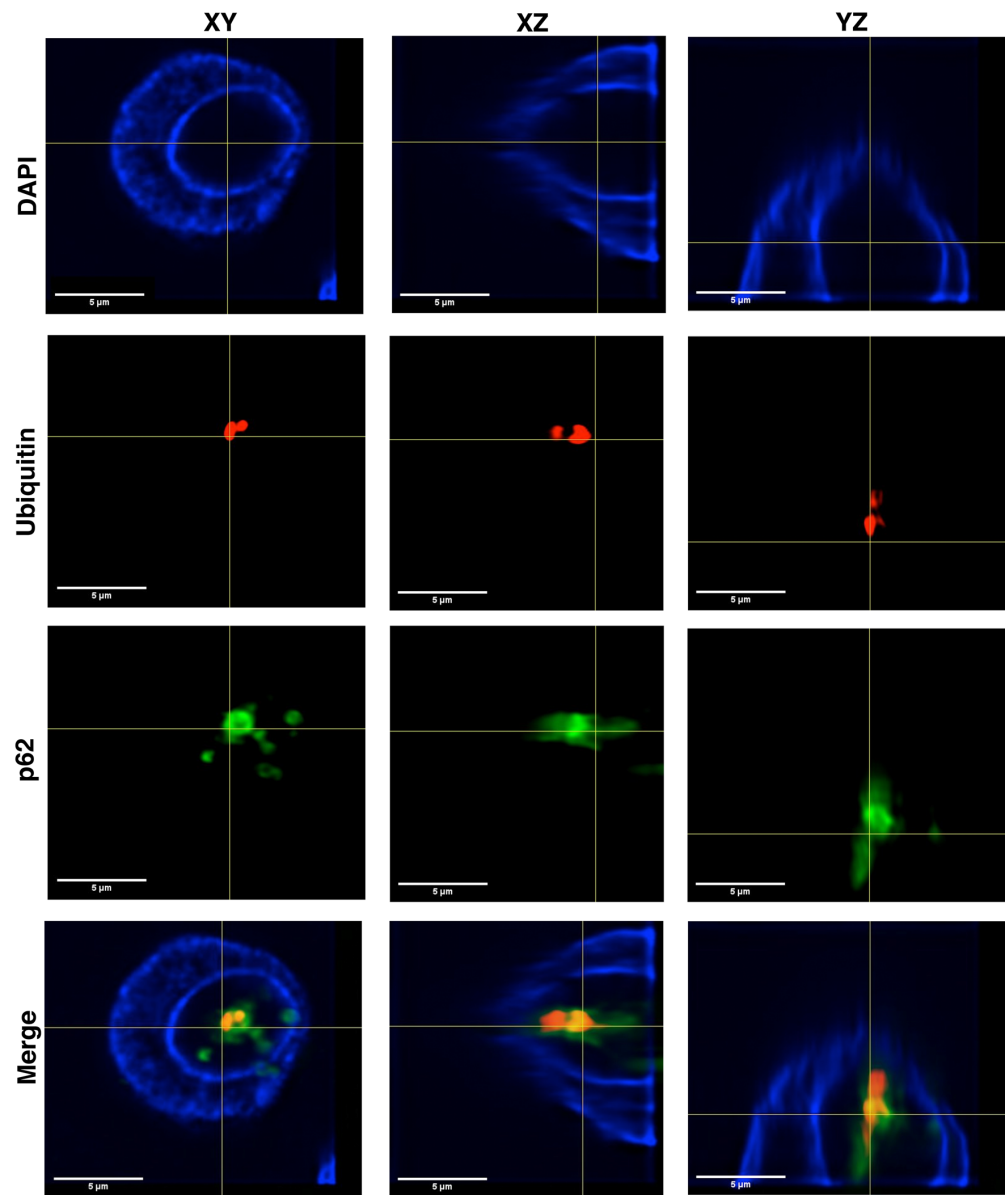
Three-dimensional imaging of double immunofluorescence-labelled (ubiquitin and p62) tissue sections in X-, Y- and Z-axis. A total of 152 optical sections of 0.1 µm were imaged and 98 of them were used for 3D reconstruction. The image demonstrates a representative case with type1 NI. DAPI staining (blue) depicts the nucleus with an intranuclear inclusion, which contains the autophagy-associated proteins ubiquitin and p62. Additionally, co-localizations of ubiquitin and p62 in the same inclusion are seen, proven by the formation of the strong merged colour yellow (merged image); ubiquitin = red, p62 = green.

To investigate the role of autophagy-associated proteins within type1 NI in more detail, we performed 3D reconstruction of immunofluorescence double-labelled (ubiquitin and p62) tissue sections (Fig. 6). The top image in Fig. 6 depicts a representative DAPI-stained nucleus with a membrane-bounded intranuclear inclusion. The images below show positive immunoreactivity for the autophagy-associated proteins p62 and ubiquitin within the same inclusion. On several planes, the co-localization of ubiquitin (red) and p62 (green) is revealed within the intranuclear inclusion shown by the evolution of the merged colour yellow (merged image in Fig. 6).

**Figure 7:**
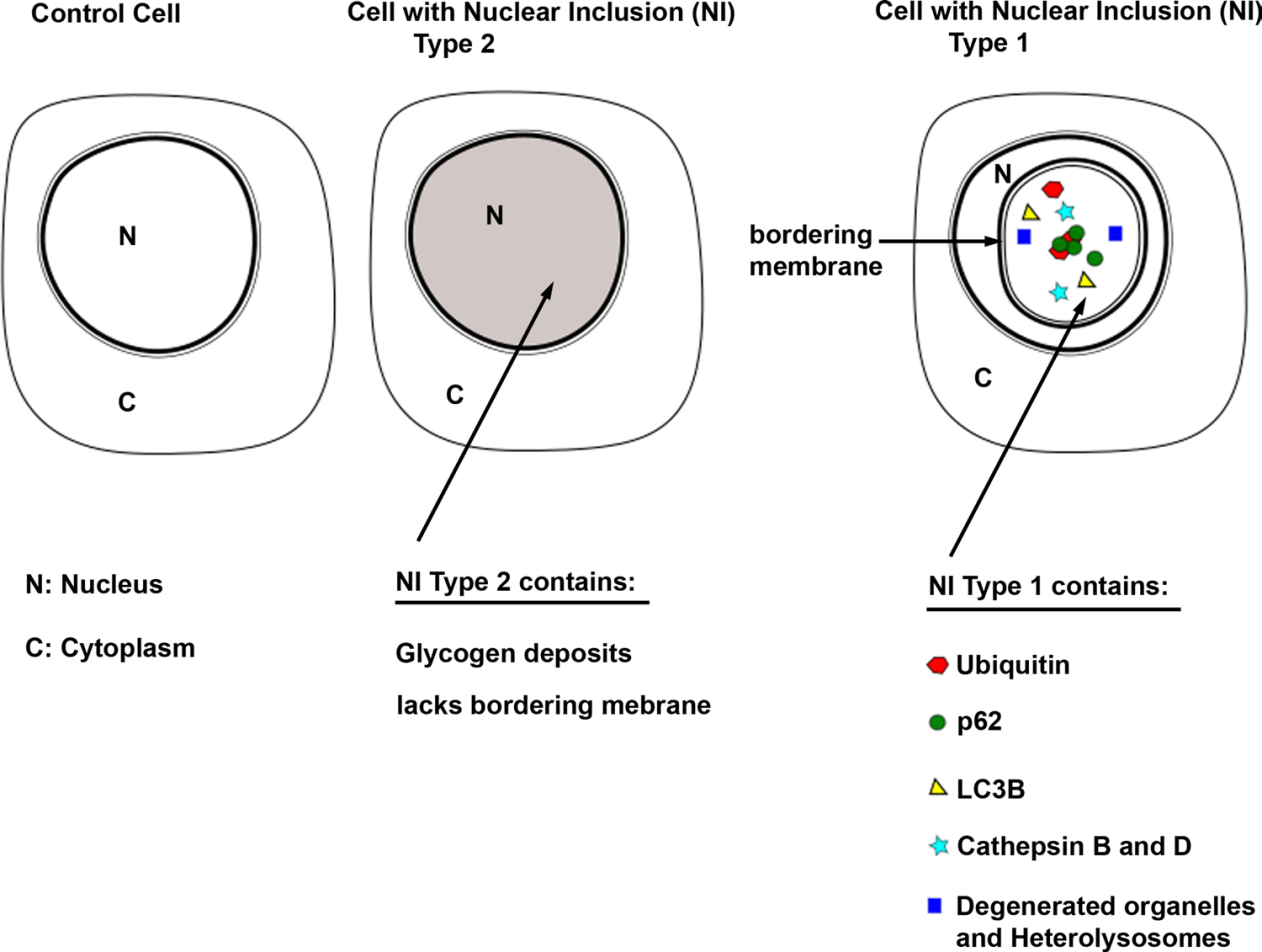
Summary figure representing our main findings. The image depicts a control cell lacking NI and two cells with NI type 1 and 2 pointing out the main characteristics of NI type1 and type2. NI type1 contain autophagy assoziated proteins, proteases and degenerated cell organelles whereby we also detected co-localizations of p62 and ubiquitin in the same NI.

Additionally, we prepared a summary figure (Fig. 7), which presents the most important results of our study.

## Discussion

As the prevalence of NAFLD is rapidly rising in industrialized countries and because of its association with diabetes and progression to advanced forms such as NASH, it becomes more important to clarify the factors driving its progression. Nuclear vacuolation is often observed in NAFLD^11,28–30^ but studies examining the shape and content of these inclusions in detail are lacking. Further, there are no investigations in NAFLD regarding biological function of these inclusions and a possible association with progression. Nuclear inclusions (NI) are generally considered as a morphological feature with no significant biological function.

We analyzed these NI in detail and detected two morphologically different NI in NAFLD: first NI delimited by the nuclear membrane (designated as “type1”) and secondly NI without limiting membrane (designated as “type2”) which, appear empty in HE and are known as glycogenated nuclei^5,6^. In the literature also two types of NI are documented; briefly, inclusions without limiting membrane, serving for accumulation of virus particles and glycogen and on the other hand membrane-bounded inclusions^31,32^. This is in line with our results, however, membrane-bounded inclusions are mostly described as pseudoinclusions representing invaginations of cytoplasm into the nucleus^31,32^.

In contrast, we demonstrate by 3D reconstruction of isolated hepatocyte nuclei (Fig. 4 and Supplementary Movie 1, Supplementary Digital Content 1) that these type1 NI are completely enclosed by the nuclear membrane and thus are located within the nucleus with no contact to the cytoplasm. Our ultrastructural investigations (TEM) of type1 NI verified the nuclear membrane as the origin of the limiting membrane (Fig. 3A,C). We documented accumulations of degenerated cell organelles and heterolysosomes in these NI, which were considerably less in the surrounding cytoplasm (Fig. 3A,C).

Similar observations were made by Kleinfeld et al. who described that degenerated organelles such as rough ER and mitochondria are more likely to occur in NI if the opening of the invagination is occluded^33^.

Thus, we can exclude that these type1 NI are mere invaginations of cytoplasm. Leduc et al. showed by TEM analysis inclusions in HCC bordered by a double membrane originating from the nuclear envelope^18^. They stated these inclusions to be an invagination of the nuclear membrane with a remaining opening to the cytoplasm of varying size. A later closure of the opening would lead to formation of a completely closed intranuclear inclusion without any contact to the cytoplasm. Nonetheless, some invaginations may not close.

Taking together the published TEM observations and our data, it is very likely that the inclusions are initially formed by invaginations, later the connection to the cytoplasm closes with the result that the invaginations/inclusions are completely localized in the nucleus.

In addition to the ultrastructural differences between type1 and type2 NI both types also differ in their immunohistochemical profile. In type1 NI we detected autophagy-associated proteins and proteases typically located in lysosomes, whereas type2 NI were negative for these proteins. The lysosome proteases cathepsin B and D together with the presence of heterolysosomes in TEM are suggestive for an active proteolytic activity in type1 NI. This proteolytic activity could also explain the nearby degenerated cell organelles, which we and others have observed in NI^20,21,33,34^. This hypothesis is supported by the identification of autophagy related proteins such as LC3B, p62, ubiquitin and cathepsins in NI. The spatial co-localization of p62 and ubiquitin within the same inclusion suggests a proteolytic process similar to autophagy. However, the canonical autophagy takes place in the cytoplasm but not in the nucleus.

We observed both NI types in NAFL and NASH but in NASH the number of type2 was about 3 times higher than that of type1. Both types were significantly more frequent in NASH than in the control-group without steatosis (*P* ≤ 0.001). This indicates that disease progression impacts the presence of NI regardless of the type of inclusion.

Further, the number of type1 NI was significantly higher in NAFL than in the controls (*P* = 0.016) and type1 NI were significantly more frequent in NASH than in NAFL (*P* = 0.012). Spearman correlations showed significant positive associations between the number of NI for type1 and type2 and the disease progression (Spearman`s rho = 0.659 and rho = 0.428, *P* ≤ 0.000). However, we have to consider that the number of NI was too small for a possible application in diagnostics. Nevertheless, our study shows that there is a significant correlation between the number of NI and disease progression; this is also consistent with the literature. Though NI can be observed both in normal and neoplastic cells^6,32,33^, they are more frequently found in neoplastic cells^20^ and are associated with clinical conditions such as diabetes, inflammatory, arteriosclerotic and neoplastic lesions, obesity and NAFLD^8,9,35^. It is documented that NI can be caused by damaging external influences, for example NI induction was observed in mouse livers after colchicine application and in kidney epithelia after chronic lead poisoning and X-ray irradiation^17^. Studies of De Oliveira et al. in a NAFLD/NASH-HCC zebrafish model showed that glycogen accumulation/ballooning degeneration were associated with inflammation in the liver and with cancer progression^36^. Thakur et al. studied in a mouse model of viral hepatitis murine hepatocytes exhibiting nuclear inclusions^37^. They found that the presence of NI correlated with oxidative stress and cellular proliferation.

We found that the percentage of type1 NI with immunoposivity for p62 and cathepsin B was higher in NASH than in the controls without steatosis. The association of hepatocellular carcinogenesis with p62 is documented^38–41^. In mouse HCC models it was shown that p62 is required in progression from premalignancy to malignancy; high p62 expression protected HCC-initiating cells from oxidative stress-induced death^41^. In addition, p62/SQSTM1-positive aggregates in liver tissue contributed to progression in NASH and HCC^38^ and p62 induced reactive-oxygen-species production and promoted hepatocarcinogenesis by an amplification of inflammation^39^. The involvement of cathepsin B in hepatic injury and fibrogenesis is also documented^42^. Canbay et al. observed that cathepsin B inactivation attenuates tissue damage in the hepatocytes of mice. Since we detected a higher percentage of cathepsin B immunopositive NI in NASH than in the controls we assume that also proteolytic proteases play a role in disease progression.

In our study, we were able to clearly define two different types of NI in NAFLD, which can be distinguished in terms of morphological features, shape, and content. Thus, we suggest that these type1 NI are not simple invaginations but probably play a role in autophagy and proteolytic processes. We have established reliable criteria for distinguishing the inclusions in NAFLD that accumulate glycogen and virus particles from the inclusions enclosed by the nuclear membrane. Intriguingly, NI gained in number with disease progression and both types were significantly more frequent in advanced NAFLD than in cases without steatosis. We suggest that the occurrence of intranuclear inclusions play a role in NAFLD progression.

## Materials and methods

### Patients

This study incorporated 77 patients. Of these, a total of 58 were morbidly obese patients (40 females, 18 males, median age 40.5, range: 20–67 and median BMI 51.7, range: 27.4–78.2) undergoing surgery in a centre for bariatric surgery; this study group consisted of 23 patients with steatosis (NAFL) and 35 with non-alcoholic steatohepatitis (NASH). According to the guidelines of the National Institutes of Health (NIH), the indication for bariatric surgery was established (BMI ≥ 40 kg/m^2^ or ≥ 35 kg/m^2^, plus co-morbidities) as described before^3^. The selection criteria for the patients were based on the same system as described in our earlier study^3^. Briefly, patients with excessive alcohol consumption (> 20 g/day in males or > 10 g/day in females) indicating alcoholic liver disease were excluded. The surgeon’s choice—i. e. adjustable gastric band; Roux-Y; or gastric bypass surgery—was in accordance with current guidelines adapted to the patient's clinical conditions and co-morbidities, and on the basis of clinical experience; during the procedure wedge liver biopsies were taken^3^. A control group of 19 organ donors (9 females, 10 males, median age 45, range: 1–80), without steatosis, had a median BMI of 23 (range: 13–44) kg/m^2^. Fatty infiltration of the liver was totally absent as all organs were dedicated for transplantation.

The diagnostic criteria for NASH have been discussed contradictory^43^. Briefly, the activity of NAFLD can be quantified according to NAFLD-activity score (NAS), as established by Kleiner et al.^44^ or according to the FLIP algorithm (fatty liver inhibition and progression) proposed by Bedossa et al.^45^. We used the FLIP algorithm by Bedossa for the classification of liver injury in morbid obesity since it allows a more accurate distinction from NAFL to NASH. Based on the histological characteristics “steatosis”, “ballooning of hepatocytes” and “inflammation”, slides were classified as “no steatosis”, “NAFL” or “NASH”. The control group showed no steatosis. More detailed information on the characteristics of the patients is given in Table 2.

Liver samples of each patient were retrieved from the files of the Institute of Pathology, University Hospital of Essen, Germany. In all cases standard processed formalin fixed and paraffin embedded (FFPE) material was stained with HE and immunohistochemical staining was performed according to institutional standards as described before^20^. From each patient an informed consent was received. The study was conducted in conformity with the Helsinki Declaration of 1975 and was approved by the Ethics Committee of the University Hospital Essen (reference number: 09–4252).

### Sample evaluation and immunohistochemistry

Two observers (HAB and JK) evaluated the HE stained slides and diagnosed NASH according to the FLIP algorithm by Bedossa et al.^45^. The NI were counted in the whole slide while total cell count was determined by DAPI staining. From each paraffin block, additional Sections (3–5 μm) were cut, dewaxed and pretreated. Immunohistochemistry was performed as described previously^20^; immunostainings of p62, ubiquitin, LC3B, cathepsin B and cathepsin D were carried out on an automated staining device (Dako Autostainer, Dako, Glostrup, Denmark). The antibodies used were: anti-p62 (#sc-28359, Santa Cruz Biotechnology, CA, USA; diluted 1:1,000 for 30 min at RT), anti-cathepsin B (#sc-6490-R, Santa Cruz Biotechnology; diluted 1:500 for 30 min at RT), anti-cathepsin D (#sc-6486, Santa Cruz Biotechnology; diluted 1:6,000 for 60 min at RT), anti-ubiquitin (#Z0458, Dako; diluted 1:1,000 for 30 min at RT) and anti-LC3B (#3868, Cell Signaling Danvers, MA, USA; diluted 1:20 for 60 min at RT). Negative controls were included in every run and incubated with non-immune immunoglobulin in the same concentrations but instead of the primary antibody. One observer (JK) evaluated the immunohistochemical stains and examined the immunostainings within the nuclear inclusions. Nuclear inclusions were classified as positively or negatively stained. Additionally, tissue sections were examined for lamin AC (nuclear membrane marker) immunoreactivity in the NI by immunofluorescence studies; briefly the primary antibody anti-lamin AC (#ab193904, Abcam, Cambridge, UK; diluted 1:50 for 60 min at RT) was labelled with the secondary antibody Alexa Fluor 488-conjugated chicken anti-rabbit IgG (#A21441, Thermo Fisher Scientific, Waltham, MA, USA; diluted 1:100 for 60 min at RT). DNA staining was performed with DAPI (Sigma-Aldrich, Steinheim, Germany) and after this cells were mounted in anti-quenching medium (Vectashield; Vector Laboratories, Inc. Burlingame, CA, USA). Image analysis was performed as described before^20^. Accumulation of glycogen in the NI was checked by PAS staining in combination with PAS-Diastase to proof glycogen infiltration.

### Transmission electron microscopy (TEM)

Ultrastructural analysis of NI was performed as described previously^20^. Briefly, for TEM, fresh liver tissue was taken as a wedge biopsy during bariatric surgery. It was fixed in 2% glutaraldehyde in 0.1 M cacodylate buffer (cB), pH 7.3 for 4 h at room temperature (RT), then washed in cb, post-fixed in 1% osmium tetroxide in cb, dehydrated in a graded series of alcohol and embedded in epoxy resin. Semi-thin sections were stained with basic fuchsin and methylene blue to specify capable blocks for ultrathin sections to mount on copper grids. The ultrathin sections were contrasted with uranyl acetate (1%) and lead citrate (0.4%) and examined using a Zeiss TEM 902A (Zeiss, Oberkochen, Germany). We used a slow-scan-CCD camera and the ITEM 5.2 software (both Olympus Soft-imaging-Systems, Münster, Germany) for digital image acquisition.

### 3D reconstruction of immunofluorescence-labelled isolated nuclei

An accurate location of an inclusion can only be retraced by scanning the whole nucleus. Thus, we accomplished 3D imaging on isolated hepatoycte nuclei as previously reported with minor modifications^20^. For immunoflouorescence nuclei of representative samples were isolated. Paraffin Sections (60 μm) were deparaffinized using xylene in a 1.5 ml reaction tube. After discarding the supernatant, the pellet was rehydrated in 99%, 96% and 70% ethanol and the supernatant was discarded. The pellet was washed in Target Retrievel Solution pH9 (#S2367, Dako), centrifuged and the supernatant was removed. After resuspension of the pellet in 400 µl Target Retrievel Solution pH9 and disruption by a homogenizer, Heat-induced epitope retrievel (HIER) was performed at 98 °C for 60 min and the suspension was cooled at RT for 30 min. Cells were equilibrated in a antibody diluent composite with Dako REAL Antibody Diluent (#S2022, Dako) containing additionally 2% BSA and 0.5% saponin for 30 min at RT and afterwards incubated with the primary antibody lamin AC (#Ab108595, Abcam, diluted 1:50 in the antibody diluent composite) overnight at 4°. After centrifugation and washing, cells were incubated with the secondary antibody Alexa Fluor 488-conjugated chicken anti-rabbit IgG (#A21441, Thermo Fisher Scientific) in a dilution of 1:100 in the antibody diluent composite for 60 min at RT. Nuclear staining was performed with DAPI (Sigma-Aldrich) before applying anti-quenching medium to the cells (Vectashield). Images were attained using a Leica TCS SP8 STED confocal microscope (Leica Microsystems, Illinois, USA) and were analyzed with the Application Suite X software (Leica Microsystems). The open source software Fiji (ImageJ; www.fiji.sc) was used for the 3D reconstruction and the Huygens Professional software (Scientific Volume Imaging; www.svi.nl/ContactSVI) was used for generation of the z-stacks.

### Double immunofluorescence and 3D reconstruction

Three-dimensional localization of p62 and ubiquitin was analyzed by double immunofluorescence staining and followed by reconstruction as previously described with minor modifications^20^. One-μm FFPE tissue sections were cut, dewaxed, rehydrated and pretreated with Target Retrieval Solution pH9 (#S2367, Dako) for 20 min at 97 °C.

For double labelling immunofluorescence the primary antibodies anti-p62 (#BML PW9860, Enzo, Life Sciences, NY, USA, diluted 1:250 for 60 min at RT) and anti-ubiquitin (#NB300-130, Novus, Littleton, CO, diluted 1:100 for 30 min at RT) were used. Secondary antibodies used were Alexa Fluor 488-conjugated donkey anti-rabbit IgG (#A21206, Thermo Fisher Scientific) and Alexa Fluor 594-conjugated goat anti-mouse IgG1 (#A21125, Thermo Fisher Scientific). Anti-p62 antibody was labelled with Alexa Fluor 488 and anti-ubiquitin antibody with Alexa Fluor 594. After 1:100 dilution they were incubated for 60 min at RT. DNA was stained with DAPI and image analysis was performed as described above.

### Statistics

Analyses were performed with the Statistical Package for Social Sciences (SPSS 24.0, Chicago, IL, USA). We used Mann–Whitney-U-test and Kruskal–Wallis test for continuous factors and two-sided Fisher’s exact test for categorical parameters. Additionally, Spearman's rho correlations were performed to determine correlations between the variables. All data are shown as medians with ranges presented in parentheses, if not stated otherwise; *P* ≤ 0.05 was defined as statistically significant.

## Supplementary information

Supplementary Table 1.

Supplementary Movie 1.

Supplementary Movie 2.

## Data Availability

Data is provided in the manuscript and/or Supplementary Data.
