# Supplementary material for: Characterization of two types of intranuclear hepatocellular inclusions in NAFLD
Source: Sci Rep. 2020 Oct 6;10:16533. doi: 10.1038/s41598-020-71646-y (PMC7538976; doi:10.1038/s41598-020-71646-y)
Supplement: Supplementary file 1 — Supplementary Table 1. [file 41598_2020_71646_MOESM1_ESM.pdf]

Title: Characterization of two types of intranuclear hepatocellular inclusions in NAFLD

Authors: Suzan Schwertheim, Julia Kälsch, Holger Jastrow, Christoph Matthias Schaefer, Sarah Theurer, Saskia Ting, Ali Canbay, Heiner Wedemeyer, Kurt Werner Schmid and Hideo Andreas Baba

Supplementary Table S1: Immunoreactivity for p62, LC3B, ubiquitin, cathepsin B and cathepsin D within type1 NI

	**Total sum of type1 NI**	**p62 type1 NI**	**LC3B type1 NI**	**Ubiquitin type1 NI**	**Cathepsin B type1 NI**	**Cathepsin D type1 NI**
**Diagnosis**	**Control**					
N valid cases		19	19	19	19	19
Sum of NI	21	1	5	2	1	3
Immunopositive NI %		4.76	23.81	9.52	4.76	14.29
**Diagnosis**	**NAFL**					
N valid cases		23	23	23	23	23
Sum of NI	210	11	6	6	31	26
Immunopositive NI %		5.24	2.86	2.86	14.76	12.38
**Diagnosis**	**NASH**					
N valid cases		35	35	35	35	35
Sum of NI	728	60	24	44	88	67
Immunopositive NI %		8.24	3.30	6.04	12.09	9.20
Sum of NI for all cases	959	72	35	52	120	96

Immunopositive NI % = Total sum of immunopositive type1 NI / Total sum of type1 NI %

N = number; NI = intranuclear inclusions; All shown values refer to type1 NI
